# Hygienic evaluation of the Resourcify GmbH concept for recovering raw materials from recyclable medical devices after surgery

**DOI:** 10.3205/dgkh000471

**Published:** 2024-04-17

**Authors:** Axel Kramer, Florian H. H. Brill

**Affiliations:** 1 Institute of Hygiene and Environmental Medicine, University Medicine, Greifswald, Germany; 2 Dr. Brill + Partner GmbH - Institute of Hygiene and Microbiology, Hamburg, Germany

## Abstract

In addition to emissions harmful to the environment, a significant amount of waste is generated in hospitals. In recognition of the fact that medical devices (MDs) contain valuable raw materials, such as rare earth elements, other metals, and high-quality plastics, a recycling concept has been developed. The project was examined for safety and feasibility from a hygiene point of view with sustainability in mind in order to create a reference solution for other areas as applicable.

The recycling process begins when the MDs accumulate in the surgical facility and are separated into recyclable and disposable parts. The recyclable parts are subjected to wipe disinfection and collected in closed boxes until they are taken away, while the non-recyclable parts are sent for disposal. The recyclable waste, including the transport boxes, is steam-disinfected in a fractionated vacuum process before recycling. The waste is then recycled, and the emptied transport boxes are made available for re-collection by the surgical facility.

The analysis of the overall recycling process shows that infectious risks both for the employees who collect, transport, and recycle the MDs and for the environment are neglectable.

## Abstract (de)

Neben klimaschädlichen Emissionen stellt das Abfallaufkommen die deutschen Krankenhäuser vor große Herausforderungen. Da Medizinprodukte (MP) wertvolle Rohstoffe wie seltene Erden, andere Metalle und hochwertige Kunststoffe enthalten, wurde ein Konzept entwickelt, um diese Stoffströme weg von der Verbrennung in das Recycling zu bringen. Mit der Zielsetzung der Nachhaltigkeit wurde das Vorhaben unter hygienischen Gesichtspunkten auf Unbedenklichkeit und Machbarkeit überprüft, um ggf. eine Modellösung für weitere Industrien zu schaffen.

Der Recyclingprozess beginnt beim Anfall der MP in der Operationseinheit mit Trennung in recycelbare und zu entsorgende Anteile des MP. Erstere werden der Wischdesinfektion unterzogen und in geschlossenen Boxen bis zum Abtransport gesammelt, während die nicht recycelbaren MP der Entsorgung zugeführt werden. Vor dem Recycling erfolgt eine Dampfdesinfektion der separierten Abfälle einschließlich der Transportboxen in einem fraktionierten Vakuumverfahren. Danach erfolgt die Verwertung der Abfälle und die entleeren Transportboxen stehen zur erneuten Sammlung im OP zur Verfügung.

Im Ergebnis der Analyse des Gesamtprozesses für das Recycling werden infektiologische Risiken für die Mitarbeitenden, die die MP sammeln, transportieren und recyceln, und für die Umwelt ausgeschlossen.

## Background

The greatest challenges facing modern civilization are sustainable development, the reduction of environmental pollution, and the reduction of the anthropogenic greenhouse gas effect, primarily through decreased greenhouse gas emissions with a focus on switching to renewable energies and rethinking our way we use resources and shift towards a circular economy. At present, the prevailing type of economic growth destroys more than the benefit it creates [1]. Climate change with increasing warming and the release of pollutants has resulted in an increase in deaths and diseases caused by heat waves, pollution of water, soil, and air, the emergence of vectors as disease carriers, lack of nutritious food, psychological problems, and extreme weather events [2], [3], [4], [5], [6]. Sustainable development is the only way to ensure that current generations can meet their needs without jeopardizing the opportunities of future generations [1]. 

The worldwide healthcare sector contributes 4.4% to global greenhouse gas emissions [7]. One third of the emissions are directly attributable to the facilities themselves and can be significantly influenced by them. Two thirds of emissions are generated in the supply chain and can be indirectly influenced through procurement methods (green procurement) [8]. The WHO sees great potential for savings in the healthcare sector [9]. 

In addition to emissions harmful to the environment, a significant amount of waste is generated in hospitals [10]. The waste generated, at around 1,430 kg per hospital bed, is almost three times that of a person in a private household [11]. 

Implementation of sustainable solutions includes exploiting new technologies and construction methods to reduce resource consumption, using resources responsibly including generating energy from waste, reducing environmental pollution caused by material inputs, using environmentally friendly products and processes and, as a new approach, recovering valuable raw materials, particularly from used medical devices (MDs). An ecological breakthrough will not have been achieved until the technology level of waste treatment has reached the technology level of goods production [1]. 

MDs contain valuable raw materials such as rare earth elements, other metals, and high-quality plastics. These are not yet being recycled. The Resourcify GmbH concept describes a way to change this and reuse valuable raw materials. The project is to be examined for safety and feasibility from a hygiene point of view with sustainability in mind in order to create a reference solution that can be transferred to other industries. A prerequisite for such recycling is to eliminate risks of infection for the employees who collect, transport, and recycle the MDs.

## Method

**Figure 1:**
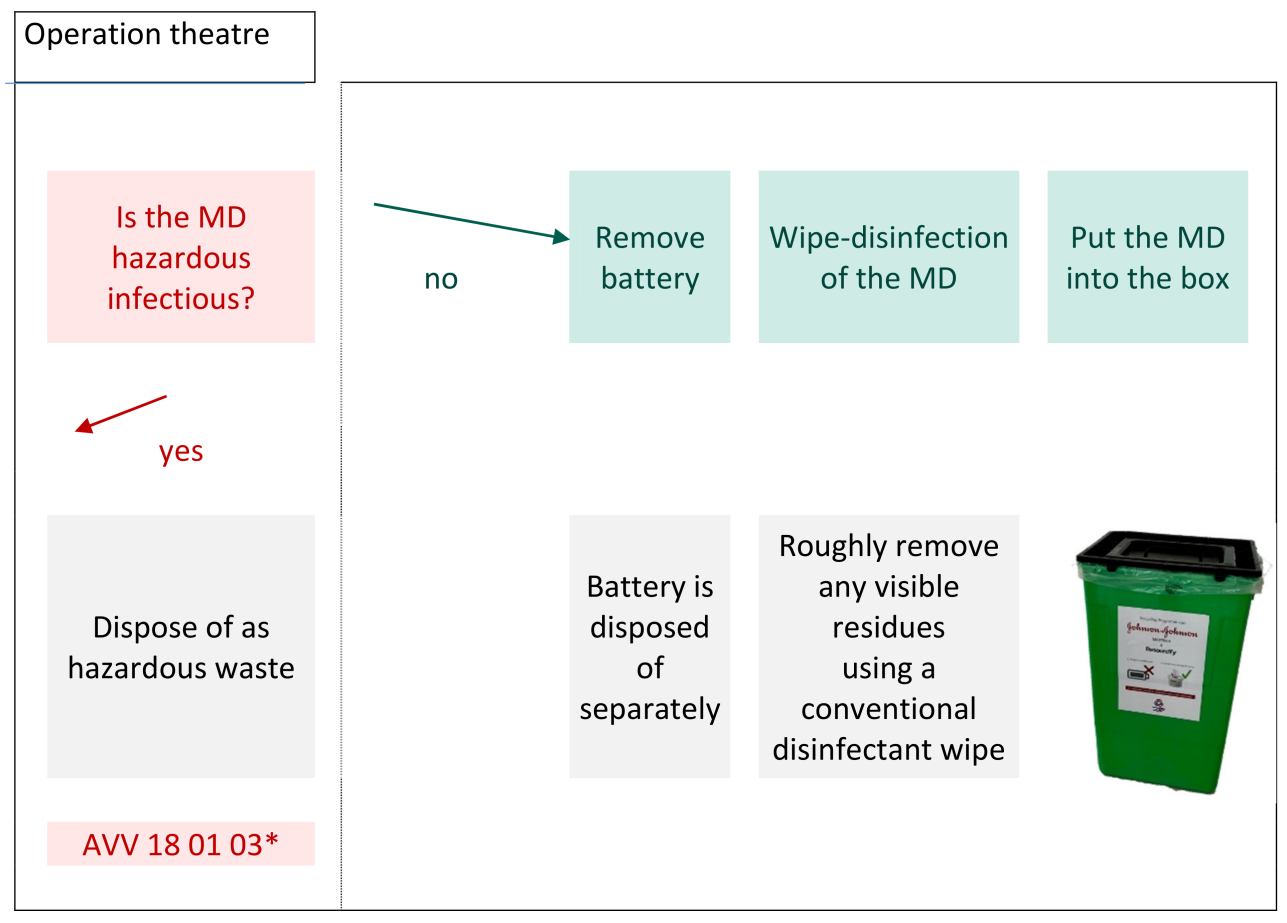
Separation into recyclable and waste parts in the surgical facility

**Figure 2:**
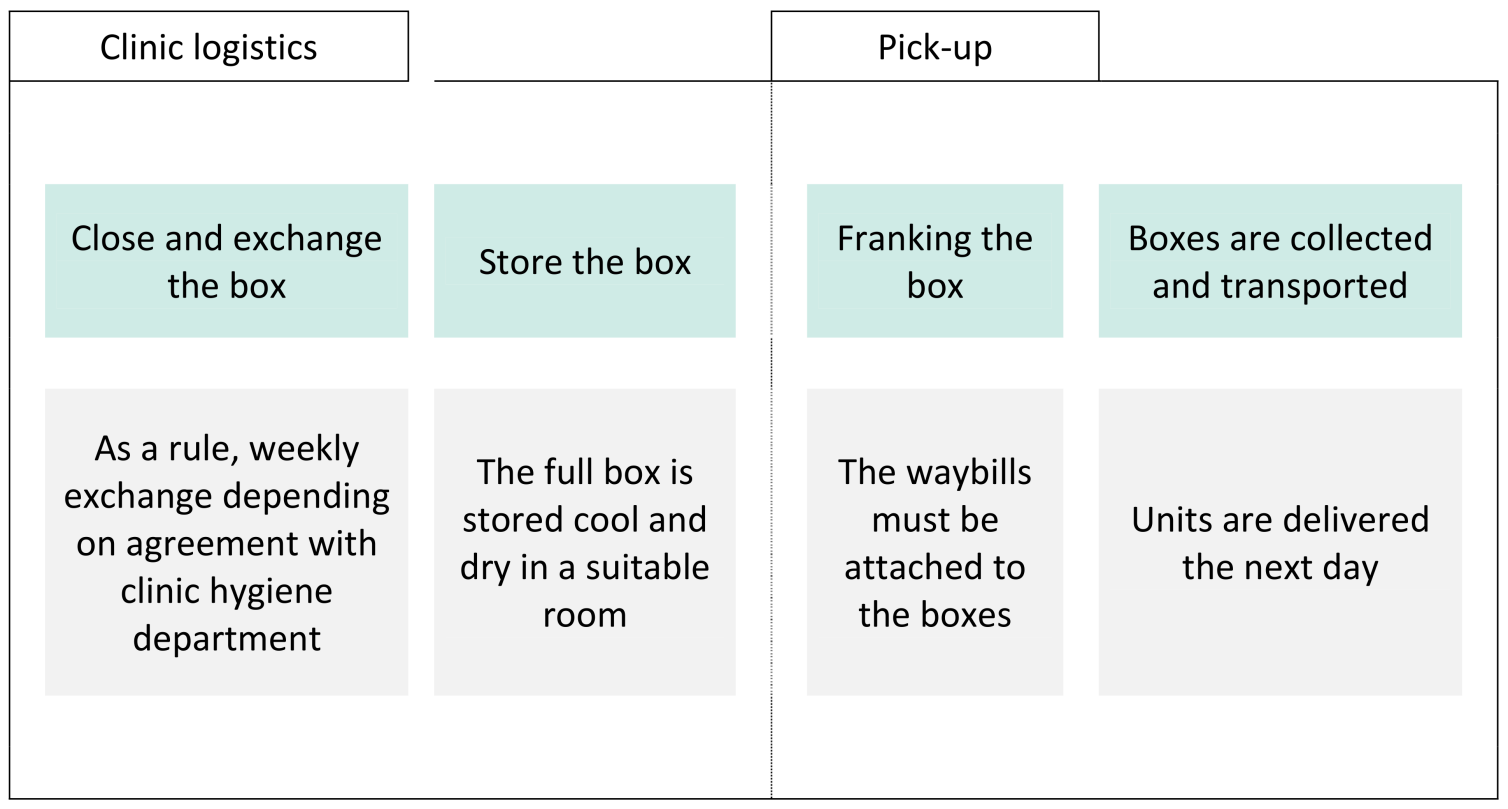
Interim storage and removal of the separated recyclable parts

The evaluation is based on the process description of the planned recycling process as well as published literature and regulation regarding infection prevention measurements. The process begins when the MDs accumulate in the surgical facility and are separated into different recycling streams (Figure 1 (Fig. 1)). The German Batteries Act (BattG) regulates the placing on the market, return and environmentally friendly disposal of batteries, which is why they must be recycled separately from the MD. A wipe-disinfection is carried out before the MD is put into the reusable recycling bin which is also equipped with a heavy-duty bag. The boxes are then sorted until transportation, followed by another cleaning process before the recycling (Figure 2 (Fig. 2)). Finally, the emptied transport boxes are available for re-collection by the surgical facility after disinfection.

## Results

### Exclusion criteria for recycling

MDs which have been in contact with patients suffering from or suspected of suffering from anthrax, gas gangrene, CJD, transmissible spongiform encephalopathy, cholera, viral hemorrhagic fever or aspergillosis are strictly excluded from this process. 

### Disinfection to disrupt potential transmission paths during separation into recyclable and disposable parts

The MD is wipe-disinfected from the outside after use on the patient. Rough surfaces or surfaces that cannot be reached directly by wiping must be soaked thoroughly to approximate the conditions of immersion disinfection. The disinfection process follows a logarithmic die-off curve [12], so it is unnecessary to wait for the surface disinfectant to act before placing it into the collection container. However, the surface disinfectant concentration is based on an exposure time of 15 minutes for safety reasons. This allows for a bactericidal, yeasticidal, mycobactericidal and virucidal spectrum of activity, since the carrier status of the patients is unknown. 

DIN EN 374 standard protective gloves with an extended cuff should be worn for personnel protection [13]. If a protective glove is visibly perforated, remove it, carry out hygienic hand disinfection with the virucidal spectrum of activity, and wait for the hands to air dry before putting on fresh protective gloves. 

### Interim storage and removal of the separated recyclable parts

In the next step, the recycling containers are closed, stored at the medical facility until approximately 4–6 boxes are ready for transport to the recycling facility (Figure 2 (Fig. 2)). It is important that the employees who close the containers perform hand antisepsis for 30 seconds after coming into contact with the lid with an active ethanol-based hand rub with a virucidal spectrum of activity. This is to prevent the spread of any infectious agents. 

The closed containers are marked with an easily removable adhesive strip that serves to seal the lids.

### Final steam disinfection

**Figure 3:**
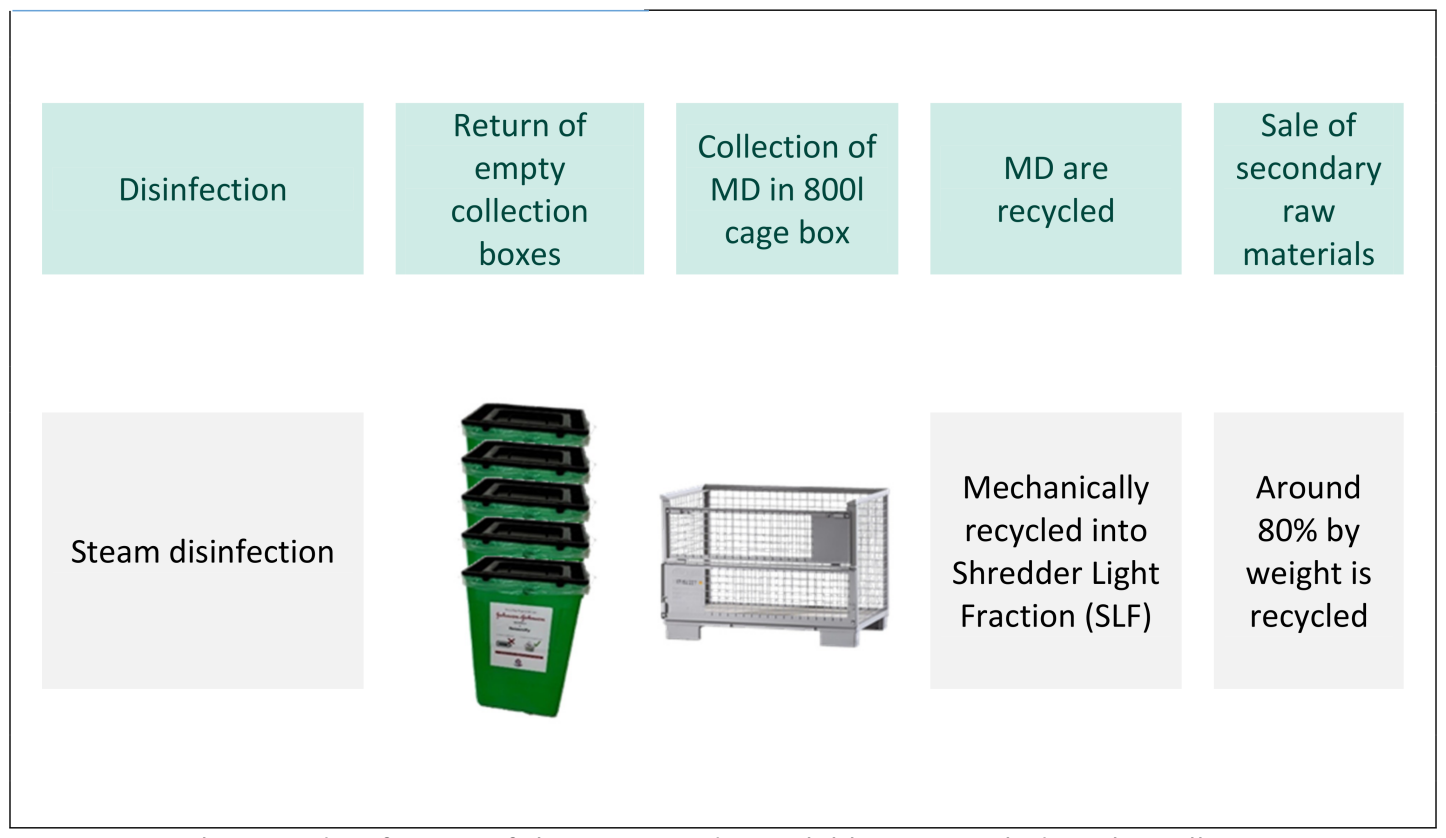
Final steam disinfection of the separated recyclable parts including the collection

In the first step, the MDs are disinfected in the recycling facility in a steam disinfector using a fractionated vacuum process at 105°C for 1 min (treatment selected from the list of disinfectants and processes tested and approved by the Robert Koch Institute [14]). The lids of the containers are opened slightly during steam disinfection so that the steam can penetrate into the containers and any elevated parts. After steam disinfection, the MDs are no longer potentially infectious and can be treated like non-medical electronic waste. The disinfected collection boxes will be transported back to the hospital and can be used for the next collection (Figure 3 (Fig. 3)). The boxes are visually inspected for damage or contamination and, if necessary, disposed of directly on site. The recycling process precludes potential reuse of the MDs on patients.

## Discussion

MDs used on patients with or suspected of having anthrax, gas gangrene, CJD, transmissible spongiform encephalopathy, cholera, viral hemorrhagic fevers or aspergillosis, pathogens that are particularly critical due to their increased chemical and thermal resistance or potential to spread are excluded and assigned to waste code 180103 [15] for secure disposal.

There is no additional hygiene risk for the first process step of wipe disinfection because even under normal disposal the MDs must be discarded and collected. The specialist staff in the surgical facility are thus familiar with the wipe disinfection technique. In addition, the electrically operated disposable instruments considered in this process are not comparable to scalpels or similar. There is therefore no increased risk of cuts or puncture wounds to be expected, which is no different from the previous use of the MDs. Incorrect disposal of packaging, instructions for use, gloves, etc. in the containers is unlikely, but cannot be completely ruled out. In this case, the artificial intelligence (AI) in the processing plant recognizes foreign materials and sorts or removes them from the recycling stream. The MDs are processed as if they were all infectious, so this does not pose a problem for the risk of infection in the process. The materials are non-critical at the end of steam disinfection in the reprocessing facility. Staff are instructed and trained accordingly to ensure an orderly procedure by complying with the process. 

The process step of wipe disinfection in the surgical facility has a lower risk of contamination than can be present in the surgical facility through contact with patients, surfaces close to patients, and inhalation. Disinfection ensures that the outside of the MDs is in a suitable condition to be safely transported. 

The first step in the overall process is simple and straightforward. Errors could occur if wipe disinfection is inadvertently forgotten. This would, however, not be a problem because the MD is discarded into a container and then sealed. Another foreseeable error could be inadvertently disposing of MDs in infectious waste. This is not a problem in terms of hygiene. It just means that the MDs would then not be recycled.

During the second process step, the intermediate storage of the disinfected MDs in sealed collection boxes, there is no risk of infection until transportation, especially as only immunocompetent persons are involved in the transport. Even in a dry state, the microbial load on or in the MDs would decrease [16]. Even if the MDs contained moisture, for example from patients’ body fluids, the adhering disinfectant residue would prevent an increase in the pathogen load. 

The closed containers are sealed, making it possible to check that the containers have not been opened without authorization, e.g. before collection or on arrival at the disinfection facility.

The steam disinfection used in the third process step kills vegetative bacteria including mycobacteria, fungi including fungal spores, as well as enveloped and non-enveloped viruses. Efficacy ranges C and D with killing of anthrax spores are not required because MDs used on patients with suspected and confirmed anthrax or gas gangrene are disposed of as hazardous waste in accordance with AS 181003 [15]. No sterilization process is required. 

After steam disinfection, both the MDs intended for recycling and the containers are no longer considered critical and can be handled without further protective measures.

## Conclusion

The recycling process is a hygienically safe process. This assumes a detailed process description, instructions, and staff training, possibly supported by a training video. There is a lower risk of infection transmission than when traveling in a crowded bus.

## Notes

### Competing interests

The authors declare that they have no competing interests.

Both authors received fees for consultation for the evaluation of hygiene risks of the process flow. The honoraria they received had no influence on the subject matter of this article.

### Authors’ ORCID

- Axel Kramer: 0000-0003-4193-2149
- Florian Brill: 0000-0001-9681-8752
